# Ein Abszess auf Abwegen

**DOI:** 10.1007/s00108-025-01863-y

**Published:** 2025-02-19

**Authors:** Hatti Seidl, Lena Thormählen, Marie Pollak, Thula Walter-Rittel, Robert Hättasch, Gerhard Hindricks

**Affiliations:** 1 Klinik für Kardiologie, Angiologie und Intensivmedizin, Deutsches Herzzentrum der Charité, Campus Charité Mitte, Charitéplatz 1, 10117 Berlin, Deutschland; 2 Klinik für Radiologie, Charité – Universitätsmedizin Berlin, Berlin, Deutschland

**Keywords:** Hirnabszess, Atriumseptumdefekt, Paradoxe Embolie, *Streptococcus anginosus*, Ösophaguskarzinom, Brain abscess, Atrial septal defect, Paradoxical embolism, *Streptococcus anginosus*, Esophageal cancer

## Abstract

Eine 69-jährige Patientin mit bekanntem Rezidiv eines Ösophaguskarzinoms wurde mit Fieber und erhöhten Entzündungswerten vorstellig. In der jüngeren Vergangenheit war ein suspekter mediastinaler Lymphknoten bronchoskopisch biopsiert worden. In Folge entwickelte sich ein Lymphknotenabszess, der über eine Fistel in die V. cava superior eine Blutstrominfektion (Erreger: *Streptococcus anginosus*) verursachte. Diese streute über einen vorbestehenden Atriumseptumdefekt bis nach zerebral, wo es zur Entwicklung multipler Hirnabszesse kam.

## Abstract (en)

A 69-year-old female patient with known recurrence of esophageal cancer presented with fever and elevated inflammatory markers. In the recent past, a suspicious mediastinal lymph node had been biopsied via bronchoscopy. Consequently, a lymph node abscess developed, leading to blood stream infection (pathogen: *Streptococcus anginosus*) via a fistula into the superior vena cava. Through a preexisting atrial septal defect, the infection also spread to the brain, causing multiple brain abscesses.

## Anamnese

Eine 69-jährige Patientin stellte sich mit Fieber (39,5 °C), Schüttelfrost und einmaligem Erbrechen in der Notaufnahme vor.

## Vorgeschichte

Vorbekannt war ein Ösophaguskarzinom (initiales Tumorstadium: T1 N2 M0, Lokalisation: mittleres Drittel, Pathologie: Plattenepithelkarzinom), welches im Vorjahr mittels definitiver Radiochemotherapie in kurativer Absicht behandelt worden war.

**Abb. 1:**
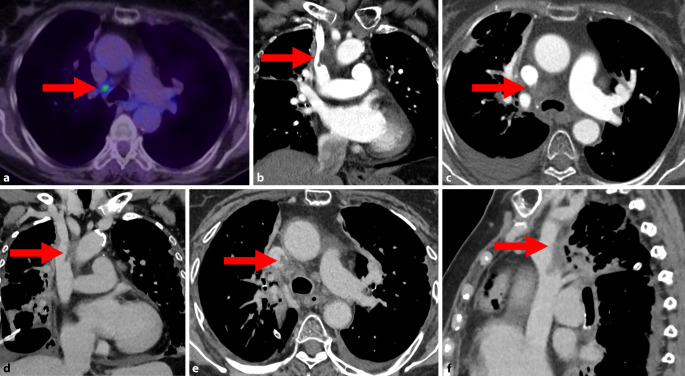
**a** Vitaler/hypermetaboler mediastinaler Lymphknoten (Station: 4R, *Pfeil*) in der PET-CT. Dies ist der Lymphknoten, der im Anschluss EBUS-gestützt punktiert wurde. **b** Erstdiagnose eines Hämatoms (*Pfeil*) nach Lymphknotenbiopsie in der CT des Thorax im koronaren Schnitt. **c–f** Verlaufs-CT mit Superinfektion des Hämatoms (**c**, *Pfeil*). Zudem flottierender Thrombus (**d**, *Pfeil*) in der VCS über der Punktionsstelle im koronaren Schnitt. Darstellung des Thrombus (axial und sagittal, *Pfeile* **e,** **f**). Zu sehen ist nebenbefundlich ein Pleuraerguss rechts. Im Verlauf wurde eine Pleuradrainage eingelegt, der Erguss ist entsprechend regredient

Jedoch wurden in der Nachsorge kürzlich endoskopisch ein Rezidiv und eine mögliche mediastinale Lymphknotenmetastase festgestellt. Der in der Positronenemissionstomographie (PET) Fluordesoxyglukose(FDG)-positive mediastinale Lymphknoten (Abb. 1a) wurde unter endobronchialer Ultraschallkontrolle (EBUS) biopsiert. Die Histologie des ösophagealen Biopsats zeigte ein Carcinoma in situ, diejenige des Lymphknotens keinen Nachweis von Malignität.

Ca. zwei Wochen nach der erfolgten Biopsie war die Patientin aufgrund einer vermuteten Pneumonie erneut stationär in der Pulmologie. In der Computertomographie (CT) stellte sich hier – neben der Pneumonie – ein Abszess des punktierten Lymphknotens dar, der über eine Fistel eine Thrombose in der V. cava superior (VCS) verursachte (Abb. 1c–f). In der Blutkultur konnte *Streptococcus anginosus* nachgewiesen werden, der Keim wurde jedoch aufgrund langer Bebrütungszeit ursprünglich als Kontamination gewertet. Eine 20-tägige (und hinsichtlich *S. anginosus* resistenzgerechte) antibiotische Therapie mit Piperacillin/Tazobactam und Vancomycin sowie eine orale Antikoagulation wurden eingeleitet, und die Patientin wurde nach fünfwöchigem stationärem Aufenthalt entlassen.

## Aktueller Befund und Initialtherapie

**Abb. 2:**
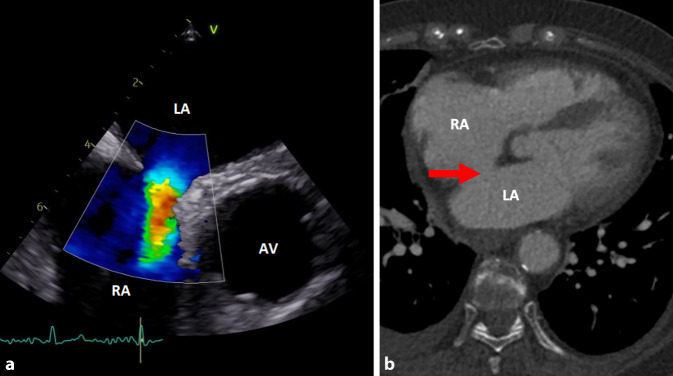
**a** Darstellung des ASD in der TEE. Der aus dem Defekt des Septums interatriale resultierende Shunt zwischen linkem (*LA*) und rechtem Vorhof (*RA*) ist sehr gut erkennbar. Rechts im Bild befindet sich die Aortenklappe (*AV*). **b** Auch in der CT lässt sich der ASD (*Pfeil*) darstellen

Im Labor zeigte sich nun – vier Wochen später – mit einer Leukozytose (16/nl; 3,9–10,5/nl) und CRP-Erhöhung (42 mg/l; < 5 mg/l) wieder eine Entzündungskonstellation. CT-morphologisch waren die mediastinale Flüssigkeitskollektion sowie die Thrombose der VCS rückläufig. Nach Abnahme von Blut- und Urinkulturen wurde eine kalkulierte antibiotische Therapie mit Piperacillin/Tazobactam und Vancomycin eingeleitet. In den Blutkulturen wurde wieder *S. anginosus* nachgewiesen, sodass nach Erhalt des Antibiogramms eine gezielte Deeskalation auf Ceftriaxon erfolgte. Die Patientin wurde unter dem klinischen Verdacht einer Endokarditis auf die kardiologische Normalstation aufgenommen. Eine transösophageale Echokardiographie (TEE) zeigte jedoch keine Vegetation, allerdings konnte ein Atriumseptumdefekt (ASD, Abb. 2) Typ II (Ostium secundum) nachgewiesen werden.

**Abb. 3:**
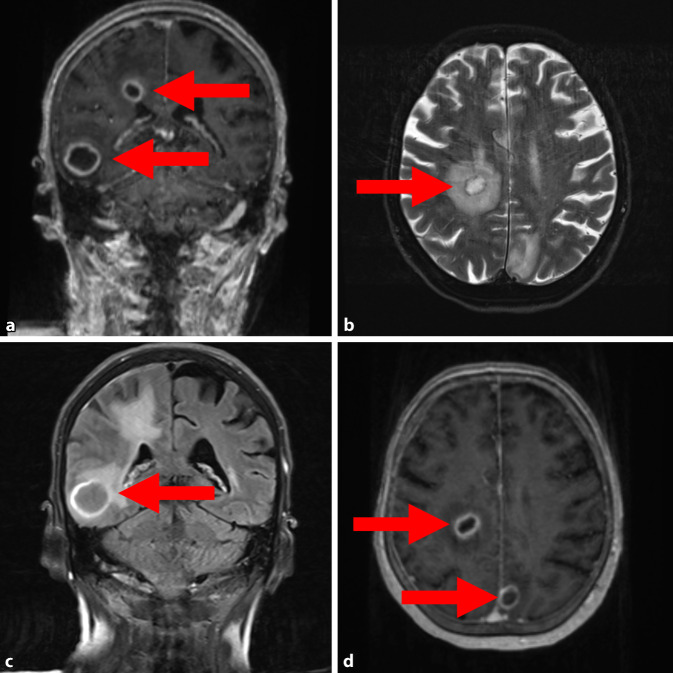
Multiple intrakranielle Abszesse (*Pfeile*) in der cMRT, die durch paradox-septische Embolien entstanden sind, jeweils markiert durch *Pfeile*. **a** Post-KM MP-RAGE koronar. **b** T2 TSE FS axial. **c** FLAIR koronar. **d** Post-KM MP-RAGE axial

Zur weiteren Diagnostik wurde bei im Verlauf neu aufgetretenen klinischen Hirndruckzeichen (Kopfschmerzen, Übelkeit/Erbrechen) eine zerebrale Computer- und Magnetresonanztomographie (cCT/cMRT) durchgeführt. Es zeigten sich mindestens acht kontrastmittelaufnehmende, intraaxiale Läsionen (Abb. 3), die radiologisch primär als Abszessformationen interpretiert wurden.

## Diagnose

Es lag eine Blutstrominfektion mit *S. anginosus* vor, welche durch den iatrogenen Lymphknotenabszess nach EBUS-gestützter Lymphknotenbiopsie mit Fistelung in die VCS entstand. Sekundär kam es über einen ASD zu paradox-septischen Embolien mit multiplen Hirnabszessen.

## Weitere Therapie und Verlauf

Es erfolgte eine längerfristige, testgerechte Antibiotikatherapie. Die Blutkulturen waren ab dem zweiten stationären Tag steril. Zusätzlich wurde die Patientin passager mit Dexamethason therapiert, worunter die Hirndruckzeichen rückläufig waren. Unter der antibiotischen Therapie sanken die klinischen und paraklinischen Entzündungszeichen weiter ab. In einer Verlaufs-TEE war weiterhin keine Vegetation nachweisbar. Eine zerebrale Verlaufsbildgebung zeigte eine teilweise Größenprogredienz der Hirnabszesse, während im CT des Thorax mit Kontrastmittel der mediastinale Abszess und der Thrombus der VCS rückläufig erschienen.

Es erfolgte die neurochirurgische Probengewinnung und Entlastung des größten, rechtstemporal gelegenen Herds. Die Biopsie bestätigte das Vorliegen eines Abszesses mit mikrobiologischem Nachweis von *S. anginosus*.

Eine chirurgische Sanierung des mediastinalen Lymphknotenabszesses war aufgrund ungünstiger anatomischer Verhältnisse nicht möglich.

Die Patientin wurde anschließend durch unsere infektiologischen Kolleg:innen weiterbetreut. Nach wenigen Tagen konnte sie bei mittlerweile bildmorphologischer Regredienz der Hirnabszesse mit einer ambulanten parenteralen Antibiotikatherapie entlassen werden. Sie ist zum Zeitpunkt der Berichtsschreibung engmaschig an die infektiologische Ambulanz angebunden. Im weiteren Verlauf ist eine erneute Vorstellung in der interdisziplinären Tumorkonferenz zur Diskussion der Therapieoptionen der malignen Grunderkrankung geplant.

## Diskussion

Dieser Fall illustriert mehrere wichtige klinische Aspekte:

### Komplikationen invasiver Diagnostik.

Die EBUS-gestützte transbronchiale Biopsie gilt als sicheres diagnostisches Verfahren, kann aber selten zu schwerwiegenden Komplikationen, wie (in etwa 0,5 % der Fälle) iatrogenen Infektionen, führen [2]. Dies unterstreicht die Notwendigkeit einer sorgfältigen Nachsorge.

### Mikrobiologische Befundinterpretation.

*S. anginosus*, hauptsächlich als Kommensale der Mundhöhle bekannt, ist ein opportunistischer Erreger [3]. Im hier vorliegenden Fall wurde der initiale Nachweis fälschlicherweise als Kontamination und der zweite Nachweis im Zusammenhang mit einer möglichen Endokarditis gewertet. Dies verdeutlicht die Wichtigkeit, mikrobiologische Befunde im klinischen Kontext zu interpretieren, insbesondere bei opportunistischen Erregern.

### Diagnostische Herausforderungen in der Onkologie.

Die Stoffwechselsteigerung im F18-FDG-PET-CT ist nicht spezifisch für Malignität. So können diverse entzündliche, granulomatöse oder auch benigne lymphoproliferative Erkrankungen zu einer mediastinalen Lymphadenopathie mit Stoffwechselsteigerung führen [4]. Daher ist die Bewertung einer Stoffwechselsteigerung im PET-CT im klinischen Gesamtkontext und zusammen mit CT-morphologischen Aspekten (Größe, Kurzachsendurchmesser und Größendynamik) wichtig.

### Rolle anatomischer Anomalien.

Der ASD spielte eine entscheidende Rolle bei der Entwicklung der Hirnabszesse durch paradoxe Embolien. Dies betont die Bedeutung einer gründlichen kardialen Untersuchung bei systemischen Infektionen. Ein Verschluss wäre nach entsprechender Vorbereitung und im Kontext einer stattgehabten paradoxen Embolie gemäß den Leitlinien denkbar [1], rückt aber angesichts der Prognose der Patientin in den Hintergrund.

## Fazit für die Praxis

- Eine sorgfältige Überwachung und Nachsorge nach invasiven diagnostischen Verfahren sind unerlässlich.
- Mikrobiologische Befunde sollten stets ernst genommen werden.
- Die interdisziplinäre Zusammenarbeit ist entscheidend, um komplexe Fälle wie diesen adäquat zu diagnostizieren und zu therapieren.
